# Functional selectivity of Receptor Tyrosine Kinases regulates distinct cellular outputs

**DOI:** 10.3389/fcell.2023.1348056

**Published:** 2024-01-08

**Authors:** Sakim S. Samad, Jean-Marc Schwartz, Chiara Francavilla

**Affiliations:** ^1^ Division of Molecular and Cellular Functions, School of Biological Sciences, Faculty of Biology, Medicine and Health, The University of Manchester, Manchester, United Kingdom; ^2^ Division of Evolution, School of Biological Sciences, Faculty of Biology, Medicine and Health, The University of Manchester, Manchester, United Kingdom; ^3^ Section of Protein Science and Biotherapeutics, Department of Bioengineering and Biomedicine, Danish Technical University, Lyngby, Denmark

**Keywords:** functional selectivity, ligand bias, ligand concentration, modelling, receptor tyrosine kinase (RTK), cell signalling, FGFR, EGFR

## Abstract

Functional selectivity refers to the activation of differential signalling and cellular outputs downstream of the same membrane-bound receptor when activated by two or more different ligands. Functional selectivity has been described and extensively studied for G-protein Coupled Receptors (GPCRs), leading to specific therapeutic options for dysregulated GPCRs functions. However, studies regarding the functional selectivity of Receptor Tyrosine Kinases (RTKs) remain sparse. Here, we will summarize recent data about RTK functional selectivity focusing on how the nature and the amount of RTK ligands and the crosstalk of RTKs with other membrane proteins regulate the specificity of RTK signalling. In addition, we will discuss how structural changes in RTKs upon ligand binding affects selective signalling pathways. Much remains to be known about the integration of different signals affecting RTK signalling specificity to orchestrate long-term cellular outcomes. Recent advancements in omics, specifically quantitative phosphoproteomics, and in systems biology methods to study, model and integrate different types of large-scale omics data have increased our ability to compare several signals affecting RTK functional selectivity in a global, system-wide fashion. We will discuss how such methods facilitate the exploration of important signalling hubs and enable data-driven predictions aiming at improving the efficacy of therapeutics for diseases like cancer, where redundant RTK signalling pathways often compromise treatment efficacy.

## 1 Introduction

**FIGURE 1:**
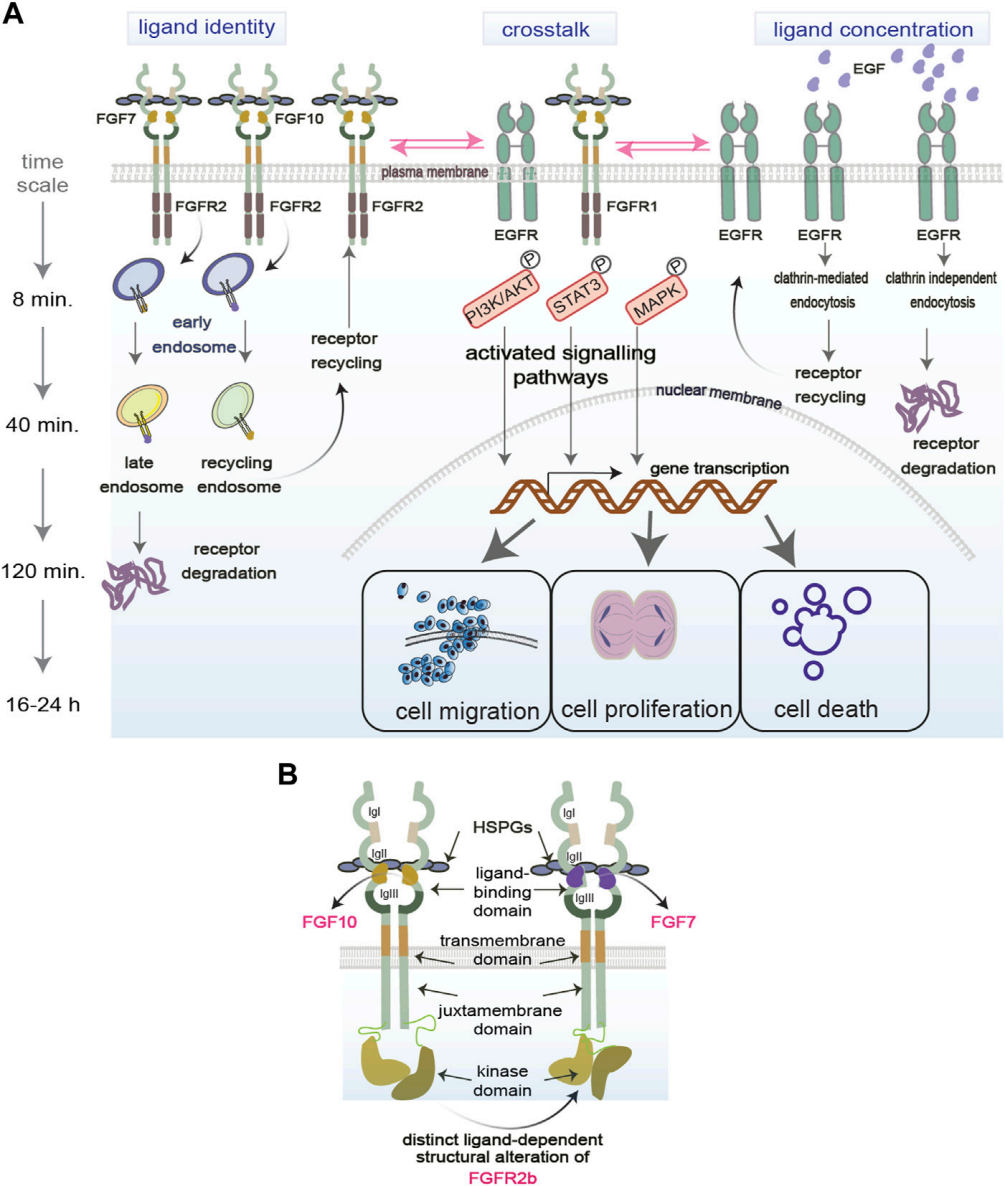
Regulation of functional selectivity. **(A)** RTKs reside on the plasma membrane and are exposed to several factors in the extracellular environment that modulate their functions. Ligand identity and concentration as well as the crosstalk of RTKs with other molecules regulate RTKs signalling (exemplified by PI3K/AKT, STAT3, MAPK) in space (early, late, recycling endosomes) and time (min vs. hours). RTK signalling specificity in turn determines the functional outcomes of the cell (proliferation, migration, death). P, phosphorylation event. **(B)** Structural regulation of functional selectivity based on the FGFR2b bound to its two known ligands FGF7 or FGF10 and to HSPGs. Different ligands induce different structural alterations that determine the PTMs and downstream signalling. Receptors can dimerise independent of ligands.

Receptor Tyrosine Kinases (RTKs), including Fibroblast and Epidermal Growth Factor Receptors (FGFR and EGFR, respectively), are plasma membrane-bound receptors that play crucial roles during development and in adult tissue homeostasis by regulating several biological processes like cell proliferation, migration, differentiation, and survival (Sigismund et al., 2017; Wintheiser and Silberstein, 2021; Ornitz and Itoh, 2022). Upon ligand binding, RTKs initiate signalling cascades which regulate such cellular responses, and which include both canonical and non-canonical signalling players (Figure 1). For instance, canonical signalling players include the MAPK, STAT3, and the PI3K-AKT signalling pathways, whereas adhesion molecules are considered non-canonical regulators of RTK signalling (Ferguson et al., 2021). Dysregulation of signalling cascades downstream of RTKs is one of the hallmarks of human diseases, including genetic diseases and cancer, and signalling molecules are among the known drug targets (Orrico, 2023). However, how the cells commit to distinct cellular outputs by regulating RTK signalling cascades in response to perturbations is still a mystery. Uncovering how signalling specificity downstream of RTKs is regulated will open new avenues for targeted therapies for patients.

## 2 Functional selectivity

The activation of differential signalling and cellular outputs downstream of the same membrane-bound receptor when activated by two or more different ligands is known as functional selectivity (Karl et al., 2020). Here, we will discuss recent biochemical, cell biological, structural, computational, and system-levels studies of functional selectivity in the context of RTK signalling.

Functional selectivity has been extensively studied for G-protein coupled receptors (GPCRs) to develop more fine-tuned drugs with less side effects (Smith et al., 2018; Onaran and Costa, 2021). However, studies on RTK functional selectivity remain sparse, except for a few advances in the last decade concerning different ligands binding to the same RTK and regulating specific signalling outputs (Karl et al., 2020). Besides distinct ligands priming the receptors for selective signalling pathways, other variables like ligand concentrations in the extracellular environment and crosstalk between RTKs or with other plasma membrane molecules can also mediate differential functional outcomes. The next sections will consider how ligand identity, ligand concentration and other molecules at the plasma membrane drive selective RTK signalling. We will also discuss how functional selectivity is regulated in space and time during receptor trafficking and by RTK structural alterations (Figure 1).

### 2.1 Ligand identity

Upon binding to the same RTK, distinct ligands initiate specific signalling cascades that lead to differential cellular outcomes (Sato and Nakamura, 2004; Francavilla et al., 2013; Francavilla et al., 2016; Watson et al., 2022) (Figure 1A, on the left). For example, stimulating FGFR2 and EGFR with their cognate ligands regulates distinct cellular outcomes like cell migration, cell proliferation, or cell survival (Francavilla et al., 2013; Francavilla et al., 2016; Watson et al., 2022). This biased signalling due to the binding of distinct ligands to the same receptor was also observed in other RTKs, such as in Insulin receptor family (Bareja et al., 2018). Also different isoforms of ligands can differentially regulate cell signalling, as shown for the two FGF8 isoforms FGF8a and FGF8b, which induces proliferation and differentiation, respectively (Sato and Nakamura, 2004). How ligand identity affects such diversity of signalling outputs is still unknown. Several mechanisms have been suggested, including distinct binding affinity of each ligand to their cognate RTKs; regulation of receptor localisation and post-translational modifications (PTMs); and assembly of specific signalling complexes (Olsen et al., 2006; Zhang et al., 2006; Francavilla et al., 2013; Francavilla et al., 2016) (https://www.biorxiv.org/content/10.1101/2023.09.13.557663v1). For instance, using quantitative phosphoproteomics to analyse global changes of FGFR or EGFR signalling in response to their distinct ligands, it has been shown that distinct PTM profiles on the receptors or on their signalling adaptors drive the assembly of signalling complexes to regulate receptor localization and ultimately cell fate (Francavilla et al., 2013; Francavilla et al., 2016; Smith et al., 2021; Watson et al., 2022). As the dysregulation of the balance between cellular outputs is a hallmark of different diseases, including cancer (Hanahan, 2022), functionally selective RTK signalling holds the promise for the development of improved cancer therapeutics. Therefore, more studies need to be conducted on ligand-receptor relationships through the lens of functional selectivity.

### 2.2 Ligand concentration

Regulation of cellular fate, particularly during development, depends on the concentration gradient of morphogens, including ligands for different RTKs (Stapornwongkul and Vincent, 2021; Yang et al., 2023). Several studies suggest that concentration-dependent signalling is regulated by a switch-like mechanism, whereby cells respond to extracellular cues only past certain concentration thresholds (Greenfeld et al., 2021; Johnson et al., 2021; Thiemicke and Neuert, 2023). This has been shown for BPM and FGF signalling during early phase of development (Serls et al., 2005; Greenfeld et al., 2021). Consistent with this idea, altering the concentration of the ligands for FGFR or EGFR can “switch” one signalling pathway or cellular outcome to a different outcome (Sato and Nakamura, 2004; Sigismund et al., 2013; Zinkle and Mohammadi, 2018) (https://www.biorxiv.org/content/10.1101/2023.09.13.557663v1) (Figure 1A, on the right). This explains the tight regulation of RTK ligand availability observed in the extracellular matrix under physiological conditions (Thotakura et al., 2019). Indeed, in the case of FGFR ligands binding with low affinity to the Heparan Sulphate Proteoglycans (HSPGs) in the extracellular matrix, an increase in affinity due to changes in the sulphation level of HSPGs, turns a shallow into a steeper one resulting in epithelial cell elongation, but not branching (Makarenkova et al., 2009). Furthermore, a switch-like mechanism provides cells with a degree of buffering, ensuring that stochastic fluctuations in ligand concentration do not initiate unwanted and yet potent mitogenic signalling cascades (Yang et al., 2023). Considering that aberrant RTK ligand concentrations play an important role in cancer (Sharpe et al., 2011), this buffer system is either lost or overloaded, leading to uncontrolled mitogenic signalling. Therefore, more efforts are necessary to uncover the molecular mechanisms integrating the responses to ligand identity and concentration, with the aim of controlling selective cellular outputs in health and diseases.

### 2.3 Other molecules

An added layer to the RTK signalling complexity is their interplay with other receptors and molecules within the ECM, such as adhesion molecules, integrins and HSPGs (Ferguson et al., 2021) (Figure 1A, in the middle). The crosstalk between different families of RTKs, for instance between FGFR and EGFR, has been described in several cancer cell models with a role in regulating the balance between cellular outputs like cell proliferation and cell motility (Kunii et al., 2008; Smith et al., 2021). This redundant mechanism regulating mitogenic signalling downstream of different RTKs may be exploited in clinical settings, where targeting multiple RTKs in concert produce better clinical outcomes than single target strategies (Quintanal-Villalonga et al., 2019; Le et al., 2021). RTKs are also known to heterodimerize and interact with other receptor classes (Latko et al., 2019; Chen et al., 2023a). For example, elucidation of GPCR-RTK crosstalk has provided with novel targets for the development of psychotherapeutics (Di Liberto et al., 2019). RTK crosstalk with integrins or adhesion molecules was shown to be essential for the control of EGFR or FGFR localization on the plasma membrane and after internalization, which in turn regulates cell migration (Caswell et al., 2008; Francavilla et al., 2009). More recently, it has been shown that also the crosstalk between FGFR2 and EGFR regulates the balance between cell proliferation and migration when the two receptors interact on the recycling endosomes after internalization (Smith et al., 2021). Together with data showing functional interaction of FGFR with cell-surface molecules like NCAM, galectin, and anosmin 1 (Gonzalez-Martinez et al., 2004; Francavilla et al., 2009; Kucinska et al., 2019), this data points to the crucial role of receptor interaction partners at different cellular compartments, such as the plasma membrane or the endosomes, as facilitators of functional selectivity.

### 2.4 Spatiotemporal control of functional selectivity

The spatiotemporal control of signalling pathways is essential for cellular functions and RTK endocytosis is among the processes regulating signalling in space and time (Sigismund et al., 2021). After ligand-induced receptor internalization via clathrin- or not clathrin-mediated endocytosis, into early endosomes (Figure 1A, on the right), the ligand-receptor pair is still able to signal. For instance, a low concentration of EGF leads to low levels of the PTM ubiquitination on EGFR, clathrin-mediated endocytosis, the recruitment of the signalling adapter protein Grb2 and sustained signalling activation (Sigismund et al., 2013). Furthermore, EGFR phosphorylates Akt and drive pro-survival signalling cascades from the early endosomes (Wang et al., 2002). Therefore, the endosomes are essential for propagating selected signalling cascades downstream of different RTKs. Depending on ligand identity, RTKs are sorted to either late endosomes for degradation into lysosomes or to the recycling endosomes to go back to the cell surface which affects downstream cellular responses and is regulated by different molecular mechanisms (Figure 1A, on the left). For instance, EGF induces EGFR degradation via the PTMs ubiquitylation and phosphorylation (Francavilla et al., 2016) and the assembly of a specific signalling complex (https://www.biorxiv.org/content/10.1101/2023.09.13.557663v1). On the other hand, FGF10 and TGFα induce phosphorylation-regulated recycling of FGFR2b and EGFR respectively, which results in a pro-migratory phenotype in epithelial cells (Francavilla et al., 2013; Francavilla et al., 2016; Smith et al., 2021). The dichotomy between receptor recycling and degradation regulates not only the fate of the ligand/receptor pair but also signalling duration and specificity. For instance, the availability as well as the retention of receptors on the recycling endosomes controls the phosphorylation of the cell cycle regulator CDK1 (Smith et al., 2021) and of the mTOR/ULK-regulated autophagy pathway (Watson et al., 2022). When the permanence of FGFR2b on recycling endosomes is impaired, the coordination of cell motility and cell cycle is lost (Smith et al., 2021), suggesting that the precise localization of FGFR2b signalling as well as the timing of such localization regulate the balance between different cellular responses in response to distinct ligands.

### 2.5 Selective signalling via RTK structural alterations

Biased signalling may be also regulated by the structural alterations of RTKs at the plasma membrane which would affect downstream signalling cascades (Figure 1B). Changes in the structural conformation of the same GPCR in response to synthetic agonists are known to affect specific signalling pathways due to unique ligand-receptor interactions (Jóźwiak and Płazińska, 2021). Similarly, altered structural conformations have been observed on RTKs in response to different ligands (Mohammadi et al., 2005; Sarabipour and Hristova, 2016; Karl et al., 2020; Huang et al., 2021). For instance, different structural conformation of the ligand-binding pocket has been reported for EGFR upon binding of its two ligands EGF and TGFα (Huang et al., 2021), thus providing a molecular mechanism underlying functional selectivity of EGFR signalling (Francavilla et al., 2016). Altered conformations of receptors may drive distinct post-translational modifications (PTMs) in their cytoplasmic domain, which subsequently can drive functionally distinct cellular outcomes, as shown for FGFR2b (Francavilla et al., 2013; Sarabipour and Hristova, 2016). Finally, strength of dimerization has also been shown to play a role in biased signalling downstream of both EGFRs and FGFRs (Freed et al., 2017; Huang et al., 2017).

Our understanding of the role of conformational changes and strength of receptor dimerization in functional selectivity has increased due to the growing use of structural biology tools such as cryo-Electron Microscopy (EM), x-ray crystallography and fluorescence-based spectroscopic assays (Freed et al., 2017; Huang et al., 2017; Zinkle and Mohammadi, 2018; Huang et al., 2021). Recent data obtained with cryo-EM indicates that the receptor dimerization process may be asymmetric and monomers of different subfamilies of FGFRs can heterodimerise to drive downstream signalling processes (Chen et al., 2023b). This data challenges our current understanding of how functional selectivity is regulated. We envision that recent advancements in cross-linking mass spectrometry (Klykov et al., 2018; O’Reilly and Rappsilber, 2018) and hydrogen-deuterium exchange mass spectrometry (Narang et al., 2020; Javed et al., 2023), both of which can be used to investigate structural alterations of proteins in a dynamic manner, will open novel avenues for structural functional selectivity.

## 3 Systems biology of functional selectivity

Traditional biochemical and structural methods have been studying functional selectivity by focusing on a small number of targets, failing to account for the complexity and the interconnected nature of the cellular signalling architecture. Systems biology, a field that deals with the emergent properties driven by the complex interactions among biological entities, provides the conceptual and computational frameworks to overcome this barrier (Yue and Dutta, 2022) (Table 1). The last decade has seen a rapid rise in the availability of omics’ data for functional selectivity due to technological advances (Dai and Shen, 2022), and the tandem rise in computational and algorithmic tools (Chen et al., 2023a; Procopio et al., 2023). For instance, phosphoproteomics provided the community with a rich source of large-scale, unbiased data on RTK signalling (Franciosa et al., 2023). A systems level approach would now focus on the global phosphorylation landscape of signalling and their roles when integrated in a system (e.g., a cell) instead of focusing only on selected kinases, PTMs, or one pathway.

**TABLE 1:** A few of the available tools for constructing and analysing cell signalling models.

Tool	Model	Description	Source
COPASI	ODE, PDE, Gillespie	Focused on biochemical networks and their dynamics. It allows both static and time-course simulations and stochastic simulation	https://copasi.org/ (Hoops et al., 2006)
GINsim	Boolean	Primarily used for modelling gene regulatory networks (GRNs). Provides user-friendly interface for robust network-based analysis (e.g., identifying steady states and network properties)	http://ginsim.org/ (Naldi et al., 2009)
StochPy	Various stochastic modelling algorithms	Particularly suitable for single cell and highly stochastic biochemical networks. Being Python-based, it can be integrated with other python-based computational tools	Python package (Maarleveld et al., 2013)
BoolNet	Boolean	Focused on simulating gene regulatory and signalling networks. Other important applications include network reconstruction from experimental data, state transition, and perturbation analysis	R package (Mussel et al., 2010)
TIMP	Partial Variable Projection	Built for analysing spectroscopic data obtained under multiple conditions and time points	R package (Mullen and van Stokkum, 2007)
MaBoSS	Boolean, Gillespie	Provides framework for model construction, visualization, simulations of mutations, drug treatments, and sensitivity analyses, and predict outcomes of specific perturbations (gain or loss of function mutations)	https://maboss.curie.fr/ (Stoll et al., 2017)
BioUML	ODE, Gillespie, Boolean	An integrative platform providing user friendly access to various powerful modelling tools. Integration with Bioconductor and Galaxy provides increased functionalities	https://www.biouml.org/ (Kolpakov et al., 2019)
SPIDDOR	Boolean	Allows analysis of Boolean networks, perform perturbation analysis and is most suitable for pharmacological investigation	R package (Irurzun-Arana et al., 2017)
CellNetAnalyzer	Boolean, ODE	It can be used for studying network dynamics, structure, and response to perturbations	https://www2.mpi-magdeburg.mpg.de/projects/cna/cna.html (Klamt et al., 2007)
PyBoolNet	Boolean	Various graph-based algorithms can be implemented for investigating network properties. It is integrated with other well-established Python packages for network analysis and visualisation	Python package (Klarner et al., 2017)
CellDesigner	ODE/PDE	CellDesigner can be used to simulate and draw biochemical networks. It is seamlessly integrated with other tools due to its support for SBML (Systems Biology Markup Language) format and SBW (Systems Biology Workbench) compliancy	https://www.celldesigner.org/ (Matsuoka et al., 2014)
BoolSi	Boolean	BoolSi can be used for simulating Boolean networks, enabling analysis of network behaviors, manipulation of node states, and exploration of conditions affecting network states	Python package https://openresearchsoftware.metajnl.com/articles/10.5334/jors.308
CellNOpt	Boolean, Fuzzy, PDE/ODE	Cell-specific models can be created by training high throughput biochemical data against previously known signalling pathways	R package (Gjerga et al., 2020)

One way in which systems biology enables us to study functional selectivity is by building complex networks that can model a particular system (Barabási and Oltvai, 2004; Ma’ayan, 2011). In fact, network science has proved to be a valuable tool in describing and predicting protein-protein interaction networks (Chen et al., 2008; Kovács et al., 2019), gene regulatory and metabolic networks (Lacroix et al., 2008; Emmert-Streib et al., 2014), and in inferring the role of PTMs (Watson et al., 2021; Leutert et al., 2023), among others. By identifying network substructures that are preferentially activated by a particular ligand, network analysis can play a crucial role in studying functional selectivity. For example, building protein interaction networks can uncover novel interaction partners under functionally selective conditions, including cell perturbation with different ligands (Kovács et al., 2019).

Systems biology also provides tools to build mathematical models from large-scale omics’ data. For example, protein co-expression patterns can be used to infer regulatory relationships and subsequent network structure (Mayer et al., 2016). Boolean models can then be used to simulate and study dynamic effects in these networks (Hemedan et al., 2022). Since these models only allows binary states, which is often not the case in biological systems, multistate logic-based models exist that allow the modelling of multiple states (Morris et al., 2010). Other modelling methods include, but are not limited to, ordinary/partial differential equations (ODE/PDE), stochastic models or constraint-based models (see references in Table 1). However, to study functional selectivity via computational modelling of proteomics data, network/logic-based approaches or machine learning algorithms may be more appropriate, since ODE/PDE require dynamic information of signalling entities, which are not always captured by proteomics experiments, and constraint-based models are mostly suited to metabolic networks. Integrating data from different omics’ technologies provides a more comprehensive view of cellular systems, which can elucidate functional selectivity at multiple levels of a protein’s lifecycle (Chen et al., 2023b). More recently, single-cell omics’ analysis has emerged to reveal new mechanisms of functional selectivity based on cell heterogeneity, thus offering insights into how the same signal can lead to different outcomes in different cellular contexts (Ahmad and Budnik, 2023).

## 4 Conclusion and perspectives

While pharmacological investigations into GPCR ligand bias has made significant strides in recent years, research into RTK ligand bias is lagging behind. More work needs to be done to investigate the role of RTK functional selectivity in health and disease. Furthermore, the availability of large-scale data and state of the art computational methodologies, when utilised in the framework of systems biology, holds the potential to model living systems and being able to predict biological outcomes. If applied to functional selectivity, system biology will allow for rapid evaluation of different functionally selective conditions in a high-throughput manner *in silico*. This information will then be used to design targeted experiments to validate hypothesis as well as better therapeutics that can preferentially bias RTKs towards a particular cellular outcome (e.g., apoptosis in tumour cells). Research and applications of RTK functional selectivity will take inspiration from the field of GPCRs, where machine learning algorithms have been used to predict if particular chemical scaffolds are more likely to show G-protein or β-arrestin bias (Sanchez et al., 2021). As more data become available on RTK signalling and functional bias with various synthetic and natural ligands, the accumulation of data could facilitate the development of predictive tools like those developed for GPCR ligand bias. Ultimately, this heralds a new era of precision medicine, where the therapeutics are more effective with minimal to no side effects.
